# Necrotizing fasciitis secondary to *Wohlfahrtiimonas chitiniclastica* in the absence of myiasis

**DOI:** 10.1093/jscr/rjag892

**Published:** 2026-10-10

**Authors:** James Sweeney, Anas El-Mahi, Colum Keohane

**Affiliations:** Royal College of Surgeons, Dublin, D02 YN77, Ireland; Cork University Hospital, Cork, T12 DC4A, Ireland; Cork University Hospital, Cork, T12 DC4A, Ireland

**Keywords:** necrotizing fasciitis, sepsis, wound management, *W. chitiniclastica*

## Abstract

*Wohlfahrtiimonas chitiniclastica* is a rare Gram-negative bacillus associated with fly larvae, myiasis, chronic wounds, and severe polymicrobial infection. We report a 71-year-old woman with diabetes, peripheral neuropathy, severe peripheral vascular disease, and previous contralateral above-knee amputation who presented with septic shock secondary to an infected right hallux ulcer. Radiographs demonstrated soft-tissue gas extending into the distal calf. Emergency exploration revealed extensive necrosis and dishwater pus tracking proximally along the flexor hallucis longus tendon, consistent with necrotizing fasciitis. Radical debridement was performed, although major amputation was initially declined. Tissue and pus cultures grew *W. chitiniclastica* and *Enterococcus faecalis*; no larvae were identified. Despite temporary clinical stabilization with intensive care support and broad-spectrum antibiotics, the foot remained non-viable and definitive above-knee amputation was required. The patient improved rapidly following source control and was discharged to rehabilitation. This case highlights the importance of early recognition, aggressive debridement, and definitive source control for survival.

## Introduction

*Wohlfahrtiimonas chitiniclastica* was first identified in 2008 from fly larvae and has since been increasingly recognized as a cause of chronic wound infections, in particular when complicated by polymicrobial bacteraemia [1]. Reported cases, though sporadic, have typically occurred in the context of social vulnerability, self-neglect, poor hygiene, and chronic comorbid disease. Identified risk factors include diabetes mellitus, alcohol misuse, homelessness, and advanced peripheral vascular disease [2]. This case demonstrates *W. chitiniclastica* as a cause of a Necrotizing Fasciitis.

## Case report

A 71-year-old female with a medical history of Type 2 diabetes mellitus, peripheral neuropathy, vascular disease, and a previous left above-knee amputation presented to the Emergency Department with a 1-week history of worsening pain, erythema, and malodorous discharge from an ulcer overlying the right hallux metatarsophalangeal joint following a fall.

On presentation, the patient was hemodynamically unstable, with significantly elevated inflammatory markers, including a C-reactive protein of 456 mg/L (<10 mg/dl) and a white blood cell count of 14 × 10^9^/L (4.0–11.0 × 10^9^/L). Initial therapy included fluid resuscitation, antibiotics, and oxygen. Plain radiographs of the right leg revealed gas in the soft tissue of the calf, suggestive of gas gangrene and concerning for necrotizing fasciitis.

The patient underwent emergent debridement shortly after presentation to hospital. Despite concerns, discussed with the patient, that major amputation might be required, the patient did not give her consent to proceed beyond debridement; even if major amputation were deemed necessary as a life saving measure.

A substantial amount of necrotic tissue and purulent material was excised from the foot including the skin and soft tissue of the hallux, infected subcutaneous tissue on the plantar surface of the 1st–3rd metatarsals, fascia and tendons spanning the length of the foot. Exploration of the distal calf was also deemed necessary and revealed dishwater pus with thickened fascia overlying the distal part of the posterior compartment of the calf. It was evident that pus had tracked proximally along the tendon of flexor hallucis longus, consistent with necrotizing fascitits.

Extensive debridement was performed. The skin incision was extended to the mid-calf revealing apparently healthy fascia and muscle at the distal part of the gastrocnemius muscle belly. The flexor retinaculum and overlying skin were divided, to allow access to the full extent of the flexor longus tendon, creating a single large wound extending from the hallux through the medial plantar foot, via the medial ankle up to the mid-calf. The contents of the tarsal tunnel were debrided en bloc with the proximal tendons and soft tissue.

At this stage it was felt that source control had been optimized to the extent possible without major amputation, for which consent had not been given.

Multiple specimens of pus, bone and soft tissue were sent for culture and fascia for histopathology. No larvae or maggots were observed during the procedure.

The patient was admitted to the Intensive Care Unit for vasopressor support, monitoring, and treatment with IV antibiotics. The patient was initially commenced on Piperacillin-Tazobactam, Metronidazole, Clindamycin, Vancomycin with a single dose of Gentamicin, in accordance with expert microbiology advice.

*W. chitiniclastica* amongst other organisms such as *Enterococcus faecalis* were identified in the purulent fluid and tissues sent from major debridement.

The patient’s condition stabilized post-operatively both biochemically and clinically however, it was clear the foot was not viable given the extent of tissue destruction. Above knee amputation was recommended to definitively control the ongoing sepsis. Once definitive source control was performed, the patient improved quickly clinically and biochemically. Antibiotics were stopped 2 weeks post major amputation and the patient was discharged to a rehab facility.

## Discussion

*W. chitiniclastica* is a Gram-negative bacillus strongly associated with myiasis, particularly involving the larvae of *Wohlfahrtia magnifica*, an insect endemic to Southern Europe and Asia and parts of Northern Europe and has since been identified throughout the world [3].

In recent medical literature, *W. chitiniclastica* has emerged as a zoonotic human pathogen, predominantly affecting immunocompromised and socially neglected individuals. Reported patient populations include those with poorly controlled diabetes, chronic alcohol excess, homelessness, severe peripheral vascular disease, and longstanding non-healing wounds [4].

Given the characteristics of the susceptible population, it is unsurprising that infection with *W. chitiniclastica* has been associated with severe, life-threatening presentations, including necrotizing soft tissue infections and fulminant sepsis [1]. To the authors’ knowledge, this represents the first documented case within the Irish population and indeed a very small number of international cases.

Furthermore this case is interesting as the vast majority of cases are associated with myiasis and thus evidence of this is usually found in the wound in the form of maggots or larvae [4]. This was not the case with our patient and whilst the patient did have an obvious entry point in the form a pedal ulcer the typically pathognomonic maggots were not identified. To our knowledge this has only been seen in one other case, that a of a terminally ill cancer patient [2].

From a microbiological perspective, *W. chitiniclastica* presents diagnostic challenges due to its rarity and phenotypic similarity to other Gram-negative bacilli. Identification relies on advanced techniques such as MALDI-TOF mass spectrometry or molecular sequencing, which may not be routinely available in all clinical laboratories. This raises the possibility that infections caused by *W. chitiniclastica* may be under-recognized or misclassified [5].

Broad spectrum antibiotics are one of the tenets of the management of severe life threatening infections such as necrotizing fasciitis, thankfully *W. chitiniclastica* appears to be susceptible to most empiric antibiotic regimens including those started in this case [3].

In the context of current societal trends in Ireland including an ageing population, increasing social inequality, and most notably rising rates of homelessness combined with persistently high levels of excessive alcohol consumption, *W. chitiniclastica* may represent an increasingly relevant pathogen within the Irish healthcare setting and indeed the wider world’s [6].
